# Impact of ecological redundancy on the performance of machine learning classifiers in vegetation mapping

**DOI:** 10.1002/ece3.4176

**Published:** 2018-06-11

**Authors:** Paul D. Macintyre, Adriaan Van Niekerk, Mark P. Dobrowolski, James L. Tsakalos, Ladislav Mucina

**Affiliations:** ^1^ School of Biological Sciences The University of Western Australia Perth, Crawley WA Australia; ^2^ Centre for Geographical Analysis Stellenbosch University Matieland, Stellenbosch South Africa; ^3^ Iluka Resources Limited Perth WA Australia

**Keywords:** functional redundancy, machine learning, predictive modeling, predictive vegetation mapping, vegetation patterns, vegetation–environment relationship

## Abstract

Vegetation maps are models of the real vegetation patterns and are considered important tools in conservation and management planning. Maps created through traditional methods can be expensive and time‐consuming, thus, new more efficient approaches are needed. The prediction of vegetation patterns using machine learning shows promise, but many factors may impact on its performance. One important factor is the nature of the vegetation–environment relationship assessed and ecological redundancy. We used two datasets with known ecological redundancy levels (strength of the vegetation–environment relationship) to evaluate the performance of four machine learning (ML) classifiers (classification trees, random forests, support vector machines, and nearest neighbor). These models used climatic and soil variables as environmental predictors with pretreatment of the datasets (principal component analysis and feature selection) and involved three spatial scales. We show that the ML classifiers produced more reliable results in regions where the vegetation–environment relationship is stronger as opposed to regions characterized by redundant vegetation patterns. The pretreatment of datasets and reduction in prediction scale had a substantial influence on the predictive performance of the classifiers. The use of ML classifiers to create potential vegetation maps shows promise as a more efficient way of vegetation modeling. The difference in performance between areas with poorly versus well‐structured vegetation–environment relationships shows that some level of understanding of the ecology of the target region is required prior to their application. Even in areas with poorly structured vegetation–environment relationships, it is possible to improve classifier performance by either pretreating the dataset or reducing the spatial scale of the predictions.

## 1 INTRODUCTION

Vegetation maps are simplified models of vegetation complexity carrying important messages about the position of vegetation types along environmental gradients. The utility of such maps extends beyond simple descriptions and audits of vegetation patterns (vegetation types and their complexes) within an area. For instance, they are indispensable tools in land‐use and biodiversity conservation planning (Akasaka et al., 2014; Ferrier, 2002; Franklin, Woodcock, & Warbington, 2000) and serve as a major source of predictive modeling in global‐change research. Vegetation maps traditionally relied on extensive field surveys (e.g. Beard, 1975; Küchler & Zonneveld, 1988), yet these can be prohibitively costly and time‐consuming, especially when the area to be mapped is large and complex (Lee & Lunetta, 1996). Recent technological advances and remotely sensed data collection have changed the way in which vegetation maps are made and enhanced the definition of boundaries between mapped vegetation units at all spatial scales. New technologies also facilitate the production of large and complex spatial (geographical and biological) datasets that can support vegetation mapping (e.g. Farr et al., 2007; Hijmans, Cameron, Parra, Jones, & Jarvis, 2005; Viscarra Rossel et al., 2015). Modern vegetation science is also experiencing a boost through the implementation of novel data‐analytical approaches, enhancing our understanding of how the vegetation patterns formed and which environmental (or man‐induced) drivers might underpin these patterns (Blois et al., 2013; Lippok et al., 2014; Reynolds, Packer, Bever, & Clay, 2003). The combination of technological advances and improved understanding allowed the development of models to reconstruct past vegetation patterns or predict potential vegetation patterns within a region. Such models can substantially reduce the time and cost of constructing vegetation maps.

Machine learning (ML) algorithms have been shown to produce models that are accurate and robust (de Souza, Boerder, Matwin, & Worm, 2016; Dickson & Perry, 2016; Osis, Hettinga, & Ferber, 2016). In principle, a ML algorithm builds a solution (model) by examining a sample dataset and identifying features or trends. The model is then applied to an unexamined dataset to make predictions. While ML has been applied to examine a diverse range of problems (e.g. Pasolli, Truong, Malik, Waldron, & Segata, 2016; Shipp et al., 2002; Tango & Botta, 2013), there has been a recent increase in its use within the geospatial and ecological sciences. For instance, ML has been successfully applied to predict species distribution (Liu, White, Newell, & Griffioen, 2013), land‐use change (Tayyebi & Pijanowski, 2014), and hydrological regimes (Cross et al., 2015) and has also been applied to vegetation mapping across a range of spatial scales using a variety of algorithms (e.g. Bradter, Thom, Altringham, Kunin, & Benton, 2011; Munyati, Ratshibvumo, & Ogola, 2013; Pesch, Schmidt, Schroeder, & Weustermann, 2011; Zhang & Xie, 2013). When applied to vegetation mapping, ML algorithms (hereafter referred to as ML classifiers) aim to create models that depict the relationships between the vegetation types identified within an area and environmental (e.g. climate, geology) or spectral reflectance variables. Although useful maps have been produced using these methods, the performance (measured in terms of output accuracy) varies with quality of the data and the scale of the classification. The impact of data quality and scale on accuracy has received much attention (Beekhuizen et al., 2014; Ghosh, Fassnacht, Joshi, & Koch, 2014), but the influence of ecological factors, specifically redundancy (see below), on the performance of ML classifiers has to our knowledge not been investigated to date. Given that vegetation classification success is essentially a function of the vegetation–environment relationship, it is critical that we gain a better understanding of how this relationship impacts on the classification results.

The vegetation patterns and their dynamics are a result of interaction of plant species forming the vegetation cover with their environment (Götzenberger et al., 2012). The presence of each species in these complex structures is determined by their life‐history: a sum of functional traits that mediate the species response and the abiotic and biotic (interactions with other species) environment (Zobel, 1992). Niche theory predicts that each species would have a unique position along environmental gradients. However, it fails to account that some vegetation types have many species that fulfill the same (or very similar) functional role within the plant communities (Kang et al., 2015) hence the functional role of those species in a plant community is considered redundant (Walker, 1992). This means that floristically distinct communities may occur in similar positions along ecological gradients in the landscape. In such highly redundant systems, many distinct plant communities can be recognized but the relationship of these communities to their environment is unclear. In contrast, low‐redundancy systems (where few, if any, plants share responses to the factors of the ecological space) have a stronger ecological link with the environment.

In this study, we investigated the impact of the vegetation–environment relationship (considered as a surrogate for redundancy) on the performance of four ML classifiers. The performance of the ML classification models was tested by predicting (modeling) vegetation in two contrasting vegetation landscapes of Western Australia, namely (a) eucalypt‐dominated tropical savannah woodlands (characterized by low redundancy) of the Northern Kimberley and (b) temperate kwongan scrub (characterized by high redundancy) of the Geraldton Sandplains. We also tested whether data pretreatment through feature extraction or feature selection would have any impact on the model performance.

## 2 METHODS

### 2.1 Datasets

Two datasets representing the vegetation of contrasting regions in Western Australia (Mitchell Plateau and Geraldton Sandplains) were compiled and used as test cases. Each area was analyzed individually. Mitchell Plateau is part of the Northern Kimberley region (approx. 14°44′S, 125°53′E) and covers approximately 480,000 ha. The size of the Mitchell Plateau, combined with the remoteness of the region and poor road infrastructure, prevents traditional field‐focused mapping methods from being efficient. A total of 148 vegetation plots (50 m × 50 m), listing all species and estimating their percentage cover, were sampled in this region. The plots were classified into plant communities by applying the Unweighted Pair Group Method with Arithmetic Mean (UPGMA) clustering on log‐transformed data and similarity ratio as resemblance (see Mucina, Stephenson, Daniel, Van Niekerk, & Boonzaaier, 2013 for details). This classification yielded 20 floristically defined plant communities. Three communities were identified as being azonal and were consequently excluded from the modeling process. This dataset is referred to as Woodland.

Geraldton Sandplains are located 270 km north of Perth (approx. 29°49′S, 115°16′E), and the region covers approximately 121,000 ha. The sandplains are part of one of the most diverse floristic regions of Western Australia, with more species diversity of sclerophyllous shrubs than anywhere else in the state (Lamont, Hopkins, & Hnatiuk, 1982). A total of 542 vegetation plots (10 m × 10 m) were sampled in this area (Woodman Environmental Consulting, 2009). This dataset, from now on referred to as the Kwongan dataset, was classified using Beta Flexible Clustering (beta = −0.25) based on Bray–Curtis dissimilarity, with no prior data transformation applied. This classification (Tsakalos, Dršková, Hruban, Mucina, & Dobrowolski, 2014; J. Tsakalos, unpublished data) identified 24 distinct plant communities.

For both regions, rare vegetation classes were merged with their most similar class(es) at the plant community classification (called Level A) scale to ensure a minimum representation of five samples. This resulted in a reduction of the number of classes in the Woodland dataset from 17 to nine, and in the Kwongan dataset from 24 to 14. The classifier comparisons were also conducted at two broader classification scales created by grouping the Level A classes to form a group of plant communities called Level B, and then further by grouping the Level B classes into a high‐order group called Level C, to examine whether redundancy changes with classification scale. At the Level B scale, the Woodland dataset contained four classes, while the Kwongan dataset had six classes. Both datasets contained two classes at the Level C scale.

### 2.2 Predictor variables

The predictors used as input to the ML classifiers (Supporting Information Table S1) were similar for the two regions and consisted of 67 climatic variables from the BioClim database (Hijmans et al., 2005) and 12 terrain‐based layers created using the SAGA‐GIS v2.1.2 basic terrain analysis tool. The terrain variables were derived from the 1‐ARC second Shuttle Radar Topography Mission (SRTM) digital elevation model (DEM) (Farr et al., 2007). In Kwongan vegetation, 23 in situ sampled soil variables were considered. Because no soil samples were taken in the Woodland vegetation, 10 variables from the 1:150,000 (90 m resolution) National Soil Grid of Australia (Viscarra Rossel et al., 2015) were used instead. None of the vegetation plots occurred within the same cell.

### 2.3 Data treatment

Three different predicting datasets were assessed in this study: (a) the full dataset (FD) containing all predicting variables for each region; (b) a dataset containing variables derived from a feature extraction (FE) process, and (c) a dataset containing variables from a feature selection (FS) process. FE involves the construction of a new (smaller) feature set derived from the full dataset (Hira & Gillies, 2015). In this study, this dataset was constructed using the principle component analysis (PCA) tool within ArcMap v10.3. The first five principal components (PCs), which explained more than 95% of the variation in the data, were retained at all scales for both regions. FS is the process of discarding unimportant variables. The FS dataset was created using the random forest (RF) tool within Salford Predictive Modeller v8.0. A proportion (40%) of the sample data was used for (out‐of‐bag) accuracy assessment. The process started with the full set of variables, after which the importance of each variable was assessed. Subsequent models were then created by excluding less important variables. This process continued until the accuracy of the model could not be improved.

### 2.4 Quantification of ecological redundancy

Ecological redundancy depends on the strength of the vegetation–environment relationship. It can be approximated by examining the environmental variables and the quantification of their relationships using Canonical Correlation Analysis (CCA), followed by a Monte Carlo permutation test as implemented in CANOCO v.4.5 (Lepš & Šmilauer, 2003). CCA is a form of constrained ordination involving two matrices: one describing the species co‐occurrence in plots, and one comprising environmental variables for the same plots. The latter matrix constrains the ordination of plots sharing species. A Monte Carlo permutation test examines the null hypothesis that the species composition (of communities) is independent of the environmental factors. During the permutations (9,999 runs), the environmental variables were randomly assigned among plots, and then new CCA analyses were performed and assessed whether random environmental data might produce equal or better ordination structure than the ordination of the real data. An *F*‐value was generated after all permutations. If the *F*‐value exceeded 0.05, the null hypothesis was accepted.

### 2.5 Machine learning classifiers

Four ML classifiers, namely classification tree (CT), RF, support vector machine (SVM), and nearest neighbor (NN), were tested in this study.

CT was selected owing to its nonparametric nature, ease of interpretability, ability to handle multiple data types, and speed of prediction. CT proceeds through a process of recursive partitioning, which splits the training data into a series of nodes based on increases in homogeneity of the subsequent groups (Breiman, Friedman, Stone, & Olshen, 1984). The result of this process is a tree composed of nodes and terminal leaves that can easily be converted into a simple set of if‐then rules. CT outputs are easy to interpret because the resulting tree can be scrutinized to understand why a given output was generated (Chen, Wang, & Zhang, 2011). However, these trees can suffer from overfitting if allowed to grow fully without pruning (Schaffer, 1993).

Random forest is an ensemble CT classifier (Breiman, 2001; Chen et al., 2011). The principle of ensemble classifiers is that a large collection of weaker classifiers (individual CTs in this case) can be used to create a strong classifier. RF involves the construction of large number of individual trees from the training data (Rodriguez‐Galiano, Ghimire, Rogan, Chica‐Olmo, & Rigol‐Sanchez, 2012). How the trees are constructed differs from CT in that a random selection of training data is used for each tree so that each tree is trained on a different set of data. Unlike CT which considers all predictor variables at each node, RF selects a random subset of predictors and these are used to identify the best split pattern. A collection of these trees is the forest where each tree is a unique classification in terms of a random selection of predicting variables and the resulting splitting pattern leading to definition of classes. Once all trees have been constructed, the objects of the studied dataset are presented to each tree in the forest, which then predicts the class to which the object would belong to. The class that was predicted the most frequently is assigned to the unclassified data through a measure of a majority vote. The trees in RF are not pruned as the collection of all trees reduces the likelihood of overfitting. Because of its stochastic nature, RF is also relatively insensitive to noise and outliers (Breiman, 2001). However, the classification accuracy of RF is at a trade‐off with interpretability (Zhang & Wang, 2009).

SVM classifiers are widely used in land cover classification studies (Duro, Franklin, & Dubé, 2012; Zhang & Xie, 2013). SVM operates by identifying a hyperplane that separates the samples of two classes in a variable‐defined space. Finding the optimal hyperplane is challenging, because there are numerous planes that could separate the training classes (Cracknell & Reading, 2014). SVM addresses this by identifying training samples (support vectors) at the transition between two classes and identifies a plane that aligns with them. The optimal hyperplane is then identified equidistant between these support vectors (Pal & Foody, 2010). Kernels are often used to transform the feature space to improve the fitting of hyperplanes. SVMs have shown capacity for producing reliable classifications even when relatively few training samples are used (Mountrakis, Im, & Ogole, 2011). SVMs are also relatively insensitive to the effects of high dimensionality, which is beneficial when dealing with a large set of predictors (Gualtieri, 2009).

NN sorts training samples according to their similarity (distance in a feature space) to each other (Bhatia, 2010). The distances between the training data can be determined using some metrics, Euclidean distance being the most commonly applied. When data that have not been classified are presented to the classifier, the distance of an unknown sample is calculated to each of the neighboring training samples and the label (class) of the closest training sample is assigned to the unknown data. Unlike many other classifiers, NN retains all training samples during the classification process which can reduce efficiency when the size of the training set is large or when a high number of predictor variables is considered.

### 2.6 Comparison of ML classifiers

The performance of the selected ML classifiers was tested using custom software created by Myburgh and Van Niekerk (2013) based on the GDAL (GDAL Development Team, 2010), OpenCV (Bradski, 2000), and LibSVM (Chang & Lin, 2011) libraries. This software uses a 60/40 split in the observation data to create training and validation datasets, respectively, requiring a minimum of five samples per class (three for training and two for validation). Confusion matrices, which use the validation dataset to determine which samples had their class correctly or incorrectly predicted, were created for each model yielding the overall accuracy (OA) and Kappa (K) values. All datasets were iterated 30 times to reduce the influence of the random selection of training data.

### 2.7 Statistical analyses

A range of nonparametric statistical tests are recommended for comparing the performance of ML classifiers (see e.g. Garcia & Herrera, 2008). In this study, we chose the aligned rank transform (ART; Wobbrock, Findlater, Gergle, & Higgins, 2011) to perform a nonparametric factorial analysis (using ANOVA procedures) and multiple comparisons when significance is detected. This analysis was performed using ARTool (Kay & Wobbrock, 2016). The testInteractions function, which is part of the Phia module, was used for carrying out the comparisons (Rosario‐Martinez, 2015). The Holm method for *p*‐value adjustment was used as recommended.

## 3 RESULTS

**Figure 1:**
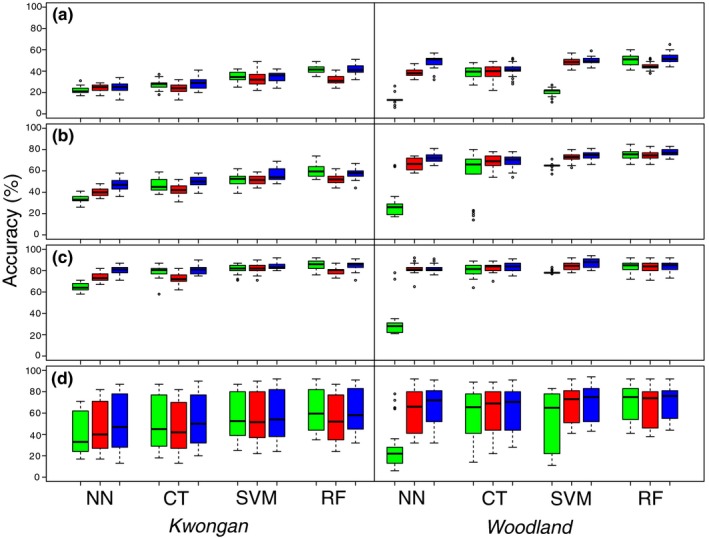
Boxplots showing classifier response to data treatment in Kwongan and Woodland at Levels (a, b, and c). The plots in section (d) represent the overall accuracy. Key: Green = FD dataset; Red = FE dataset; Blue = FS dataset; NN = nearest neighbor; CT = classification tree; SVM = support vector machine; RF = random forests

The results are summarized by classifier and scale of analysis (Levels) in Figure 1. Examples of the differences in predicted patterns at the finest scale (Level A) for each region are presented in Supporting Information Figures S1 and S2. A summary table of mean accuracy is shown in Supporting Information Table S3, while matrices showing the results of all pairwise comparisons for each region/scale are presented in Supporting Information Appendix S1.

### 3.1 Ecological redundancy patterns in the contrasting datasets

On purely statistical grounds, CCA analysis (and associated Monte Carlo permutations) showed that the vegetation patterning of the Woodland vegetation are well explained by the selected environmental variables (*F*‐value = 0.0195), while with the Kwongan vegetation, this was not the case (*F*‐value = 0.0520). In terms of ecological redundancy, we suggest that the detected vegetation–environment relationship in the Woodland is a sign of low (if any) ecological redundancy, while Kwongan is ecologically redundant.

### 3.2 Feature selection

While not all models showed improvements in accuracy (see Supporting Information Table S2), it was possible to reduce the number of predictors in all cases. This dataset contained a reduced number of 15, eight, and seven predictors for Levels A, B, and C, respectively, in Kwongan and 16, 13, and 10 for Levels A, B, and C, respectively, in Woodland. The FS dataset for Kwongan mainly comprised soil and topographic variables, while the Woodland FS dataset included some climatic, in addition to soil and topographic, variables.

### 3.3 Classifier performance

The results show that each factor (scale, region, treatment, and classifier) had a significant effect on prediction accuracy with the interactions between these factors also showing significance. The two vegetation datasets used are significantly different overall (and at each scale), with predictions in the Woodland vegetation deemed more accurate. The results, at each scale of analysis, are consequently outlined separately for each region below. Unless otherwise specified, all significant differences were found at the level of *p* < 0.001.

### 3.4 Kwongan dataset

#### 3.4.1 Level A

When the full dataset was used as input to the classifiers at Level A in the Kwongan, the accuracies of all classifiers were significantly different from each other, with RF (41%) and NN (22%) providing the best and worst accuracies, respectively. With the FS dataset, RF (42%) also outperformed the other classifiers with NN (25%) again producing the weakest models. With the FE dataset, two groups of classifiers were found, with SVM (35%) and RF (32%) returning significantly higher accuracies than CT (23%) and NN (24%). Response to pretreatment varied from making no significant difference—when either FS or FE was applied to SVM (35% each time) and NN (22%–25%), and when FS was applied to RF (41% vs. 42%) and CT (27% vs. 29%)—to significantly reducing classifier performance—when FE was applied to RF (32% vs. 41%) and CT (23% vs. 27%). Overall, the RF classifier, combined with the full and FS datasets, provided the best performance (41%, 42%) at Level A in the Kwongan dataset.

#### 3.4.2 Level B

The experiments carried out at Level B in the Kwongan showed that all classifiers produced significantly different accuracies when the full dataset was used as input. As at Level A, RF generated the strongest models (60%), while the NN classifier performed relatively poorly (34%). With both the FE and FS datasets, the classifiers formed two distinct groups with RF (FE: 52%, FS: 58%) and SVM (FE: 51%, FS: 56%) forming a group with higher accuracy than CT (FE: 42%, FS: 50%) and NN (FE: 40%, FS: 47%). Pretreatment did not make a difference to the performance of the CT or SVM classifiers. The accuracies of the NN classifications were significantly different among datasets, with FE and FS significantly improving (by 6% and 13%, respectively) performance. Overall, three classifier dataset combinations provided the best predictive accuracy, namely RF using the FD (60%) or FS (58%) datasets, and SVM using the FS dataset (56%). Compared to Level A, all classifiers performed better at Level B.

#### 3.4.3 Level C

Using the full dataset as input at Level C, NN produced significantly lower accuracies (15%–20%) compared to the other classifiers, while the differences between SVM, CT, and RF were marginal. FS had no significant impact on RF and CT, while FE reduced the accuracies of these classifiers substantially (7% and 6%, respectively). NN benefitted (FE: 9%, FS: 15%) the most from the pretreatments, but had no impact on SVM performance. Overall, the highest accuracies were achieved when the FS dataset was used as input to RF (85%) and SVM (84%).

### 3.5 Woodland dataset

#### 3.5.1 Level A

Using the full dataset as the input at Level A, RF returned the highest accuracy (50%) with SVM (20%) and NN (13%) producing the weakest models. When the FE dataset was considered, two groups with notably different accuracy statistics were noted. RF (45%) and SVM (48%) formed a group with significantly higher accuracies than CT (39%) and NN (39%), while no significant differences among classifiers within each group were noted. With the FS dataset, it was found that CT returned the lowest accuracy (42%), while the results of RF (52%), SVM (50%), and NN (49%) were on par with one another.

The treatments resulted in significant improvements to the accuracies of SVM (FE: 48%, FS: 50%) and NN (FE: 39%, FS: 49%), but had no effect on CT. The FS dataset had no effect on the performance of RF, but FE significantly reduced its accuracy (by 5%). Overall, the full dataset using the RF classifier (50%)—or the FS dataset using RF (52%), SVM (50%) or NN (49%)—performed the best at Level A in the Woodland.

#### 3.5.2 Level B

Using the full Woodland dataset as input at Level B, all classifiers returned statistically different accuracies, with RF (76%) performing the best and NN (31%) generating the weakest models. Pretreating the full dataset made significant improvements to the performance of CT (FE: 69%, FS: 69%), SVM (FE: 73%, FS: 75%), and NN (FE: 66%, FS: 72%), but had no effect on RF. Overall, the RF classifier consistently (using any of the datasets) outperformed the other classifiers (74%–77%), while the SVM classifier produced similar results when the FE (73%) or FS (75%) datasets were used as input.

#### 3.5.3 Level C

At Level C, RF (85%) and CT (81%) outperformed the other classifiers when the full dataset was used as input. NN (30%) returned the lowest accuracy. Pretreating the dataset had no effect on the accuracy of CT and RF, but significantly improved the performance of both SVM (FE: 84%, FS: 87%) and NN (FE: 82%, FS: 82%). Overall, the highest performance was obtained using the FE or FS datasets with either SVM (FE: 84%, FS: 87%) or RF (FE: 83%, FS: 84%).

In general, the Kappa values resulting from the experiments agree with the overall accuracies. However, some interesting differences were noted. For example, when the full dataset was used as input in Woodland, the Kappa values of SVM (0.02–0.08) and NN (0.005–0.03) were very low, which indicates that the models produced accuracies that are similar to what one would expect from random class assignment. While accuracy increased at Levels B and C, these classifiers were unable to return Kappa values of above 0.08 (on average) when the full dataset was used as input. However, the application of FE and FS resulted in substantial increases in reliability. For example, at Level A, the average Kappa for the SVM classifier increased by 0.34 (FE) and 0.23 (FS), while for NN, it increased by 0.26 (FE) and 0.21 (FS). These increases became more pronounced as the scale was reduced. For instance, in the case of SVM, accuracies increased by 45% from Levels A to C and 13% from Level B to C.

## 4 DISCUSSION

The results of this study show that the vegetation of the Kwongan is ecologically redundant. The hypothesis that such redundancy reduces the power of ML classifiers in predicting vegetation patterns is supported by the observations that the accuracies of the Kwongan classifications—especially at Levels A and B—were consistently lower compared to those of Woodland. It is well known that classifiers behave differently under conditions of data redundancy, however, given that the same number of features was used as input and given that the same dimensionality reduction methods were employed in both study areas, the differences in accuracy are most likely due to inherent redundancies in the environmental variables used. The significant difference in classifier performance between the regions shows that—based on the ecological relationships of the regions—we can predict the vegetation patterns of the nonredundant Woodland vegetation with greater confidence than those of the Kwongan. Although other factors may also have contributed to the differences in accuracy—for example, the classification schemes (the woodland vegetation types are better defined) and the differences in data sources (ground collected soil vs. the interpolated soil data)—the results offer evidence that ecological redundancy must have been a major driver. While different resemblance and clustering methods can change the final classification, the resulting regional systems used in this study were selected through a comparison and validation process (e.g. Tichý, Chytrý, Hájek, Talbot, & Botta‐Dukát, 2010). While different combinations were used in each region, they represent the best systems for each type.

The finding that higher accuracies were obtained when the complexity was reduced (by carrying out the experiments at coarser scales) was expected, but the way in which the classifiers responded to the FE and FS data treatments provided new insights into the impact of ecological redundancy on ML classifications. Both FE and FS have been found in many studies (Babaoğlu, Fındık, & Bayrak, 2010; Chandrashekar & Sahin, 2014; Howley, Madden, O'Connell, & Ryder, 2006) to improve classification accuracy, and it was therefore expected that predictions following these methods would be more accurate than predictions made on the full dataset. However, in the ecologically redundant Kwongan, these methods led to statistically significant classification improvements in only three scenarios, whereas in the nonredundant Woodland, they improved accuracies 12 times. Furthermore, the use of FE in the Kwongan was found to significantly reduce the performance of the classifiers four times. These findings can be explained within the context of the relationship each vegetation type has with the environment. The vegetation–environment relationship has little structure where redundancy is present and data treatments—designed to capture the variability (FE) of the environment or reduce uninformative variables (FS)—thus have little effect. An unexpected result was that the tree‐based classifiers (CT and RF) showed little response to both FE and FS in both regions. For FE, this is likely a result of the way that these classifiers create their predictions, with the recursive partitioning possibly not as strong using the transformed data.

RF unresponsiveness to FS is attributed to its use of a randomly selected subset of features during model building. Feature selection is consequently inherent in the algorithm. Although some studies (e.g. Gilbertson & Van Niekerk, 2017) have shown that RF classification accuracies can be improved using FS pretreatments, it was not the case with our data. For both regions and all classifiers, where a difference between the treatments was found in all cases FS was found to be significantly more accurate. Difference in response between the two treatments was more common in the Kwongan than in Woodland, suggesting that in conditions of redundancy, FS is more beneficial (or at least not detrimental) to classification accuracy compared to FE. We suggest that this is due to the creation of the PCs in the FE process. In this process, the input features (environmental variables) which show the greatest variation are considered (reasonably) to be more important in the construction of the new feature set (Shlens, 2014). It is important that, this process is conducted independently of the prediction targets (vegetation classes). This is, in contrast to the FS process in which the feature set, examined against our classes to determine which are the most informative in separating the classes. Given the ecological redundancy of the Kwongan, it is possible that the FE process downplayed the importance of those features which better separate the classes.

In each region, the RF and SVM classifiers consistently outperformed the other classifiers. This is consistent with the results of other studies (Duro et al., 2012; Pal, 2005; Roli & Fumera, 2001), where it was found that RF and SVM were more successful, especially under complex conditions. One can argue that, in this study, ecological redundancy contributed to complexity and that it offers an explanation why these classifiers performed better than NN and DT.

The use of ML classifiers to create robust maps of vegetation patterns is becoming more popular. However, the influence of ecological features on these classifiers is still poorly understood. The results of this study show that, although ecological complexity (i.e. redundancy) has a negative effect on classifier performance, it is not the only factor that contributed to overall performance of classifiers. The unexpectedly poor performance of NN and SVM in the Woodland suggests that the number and quality of training samples may have affected (through the addition of noise) the classifications. The finding that both FE and FS improved the performance of these classifiers in this region provides some support for this notion.

This study focussed on the application of ML to produce potential vegetation maps and as such purposefully omitted additional sources of information (such as satellite reflectance data) as this would have restricted the predictions to extant vegetation. However, it would be beneficial to examine what effect the inclusion of these datasets (either in addition to the environmental data or as sole predictors) may have on improving modeling accuracies. It is clear that more work is needed to find cost‐efficient and accurate methods for generating vegetation maps over large and complex areas.

## CONFLICT OF INTEREST

None declared.

## AUTHORS’ CONTRIBUTION

AV, LM, and PM conceived and designed the study. MD managed the collection of field environmental data. PM collated the spatial (GIS) data and performed all GIS‐related and statistical analyses. PM and LM led the writing of the manuscript, with AV providing further guidance. JT provided an unpublished vegetation classification scheme. All authors contributed critically to the drafts and gave final approval for publication.

## DATA ACCESSIBILITY

The data for this paper are lodged within Dryad. https://doi.org/10.5061/dryad.1m8tg17
