# Supplementary material for: Impact of ecological redundancy on the performance of machine learning classifiers in vegetation mapping
Source: Ecol Evol. 2018 Jun 11;8(13):6728–37. doi: 10.1002/ece3.4176 (PMC6053567; doi:10.1002/ece3.4176)
Supplement: Supplementary file 1 [file ECE3-8-6728-s001.pdf]

SUPPLEMENTARY MATERIALS

Contents

Table S1. Name and source of predictor variables 1

Table S2. Variables selected for each scale following Feature Selection. 2

Figure S1. Examples of the differences in the predicted vegetation patterns in the Kwongan between Datasets 3

Figure S2. Examples of the differences in the predicted vegetation patterns in the Woodland between Datasets 4

Table S3. Summary table of the overall accuracy and kappa values of all model iterations 6

Appendix S1. Matrices showing the results of all pairwise comparisons separated by Region and scale 7

Table S1. Name and source of predictor variables

Type of Predictor	Source	Type of Predictor	Source
**Climate**		**Soil (Woodland)**	
Bioclim 1–19		Available Water Content	
Average Preciptation (Prec 1–12)	BioClim dataset (Hijmans *et al.* 2005)	Bulk Density (Whole Earth)	
Maximum Temperature (Tmax 1–12)		Percentage Clay	
Minimum Temperature (Tmin 1–12)		Depth to regolith	Australian Soil Grid (Viscarra Rossel *et al.* 2015)
Mean Temperature (Tmean 1-12)		Depth of Soil	
		ECEC	
**Soil (Kwongan vegetation)**		pHc	
Conductivity		Percentage Sand	
Bulk Density		Percentage Silt	
Percentage Clay		Soil Organic Carbon	
ECEC		Total Nitrogen	
Effective $CaCO_3$		Total Phosphorus	
ESP			
Exchangeable Acidity		**Topographic**	
Exchangeable Aluminium		Elevation	
Exchangeable Calcium		Slope (Degrees)	Derived from SRTM DEM
Exchangeable Potassium		Aspect	
Exchangeable Magnesium		Plan Curvature	
Exchangeable Sodium		Profile Curvature	
Exchangeable Aluminium (KCl)	Ground sampled	Convergence Index	
Exchangeable Hydrogen (KCl)		Closed Depressions	
Organic Carbon		Catchment Area	
pH ($CaCl_2$)		Wetness Index	
pH (H_2O)		LS-Factor	
Percentage Sand		Channel Network Base Level	
Percentage Silt		Vertical Distance to Channel Network	
Total Carbon		Valley Depth	
Total Nitrogen		Relative Slope Position	
Total Phosphorus		Channel Network	
Water repellency			

Table S2. Variables selected for each scale following Feature Selection.
Variables are sorted by type in the order of Soil, Topographic and then climatic variables

Kwongan		
Level A	Level B	Level C
Clay (%)	Sand (%)	Bulk Density
Sand (%)	Bulk Density	Conductivity
Silt (%)	ECEC	ECEC
Bulk Density	Exchangeable Calcium	Exchangeable Calcium
Conductivity	Exchangeable Magnesium	Exchangeable Magnesium
ECEC	Total Phosphorus	Exchangeable Potassium
Exchangeable Calcium	Channel network base level	Exchangeable Sodium
Exchangeable Magnesium	Wetness Index	
Exchangeable Sodium		
Total Phosphorus		
Aspect		
Channel network base level		
Wetness Index		
**Woodland**		
Level A	Level B	Level C
Sand (%)	Silt (%)	Bulk Density
Silt (%)	Bulk Density	ECEC
Aspect	ECEC	Channel network base level
Channel network base level	Aspect	Elevation
Depth of soil	Channel network base level	Prec4
Depth to regolith	Elevation	Tmax7
Elevation	Valley Depth	Tmean3
LS-Factor	Tmin1	Tmean7
Slope	Tmin2	Tmin3
Soil Organic Carbon	Tmin4	Tmin9
Valley Depth	Tmin10	
Tmax1	Tmin11	
Tmax3	Tmin12	
Tmax7		
Tmax9		
Tmin11		

Figure S1. Examples of the differences in the predicted vegetation patterns in the Kwongan between Datasets

The predicted vegetation patterns for the Kwongan vegetation at the finest (Level A) classification scale. A: Predicted vegetation patterns using the Full dataset with the Random Forests Classifier; B: Predicted vegetation patterns using the FE dataset and the Support Vector Machine Classifier; C: Predicted vegetation patterns using the FS dataset with the Random Forests Classifier

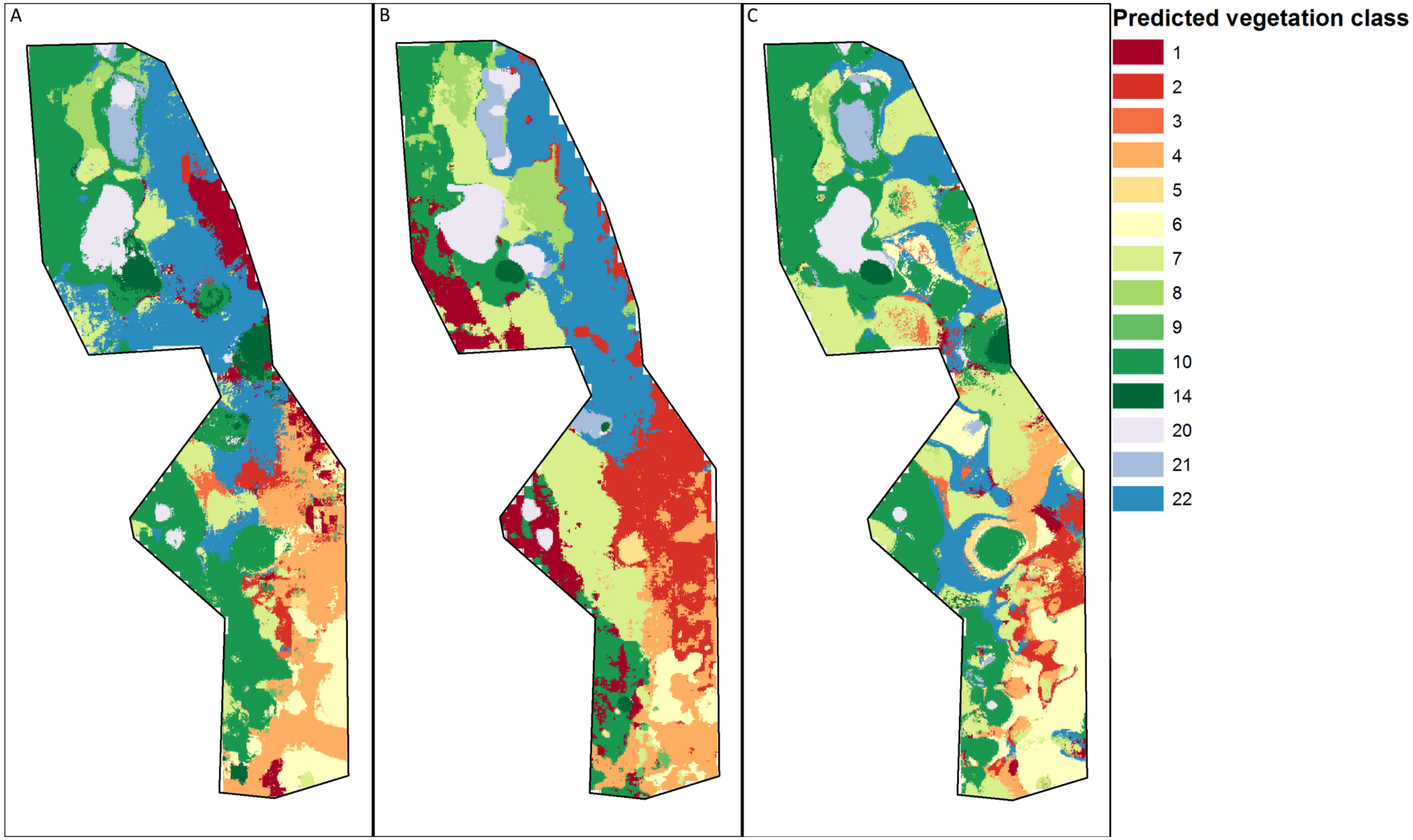

Figure S2. Examples of the differences in the predicted vegetation patterns in the Woodland between Datasets

The predicted vegetation patterns for the woodland vegetation at the finest (Level A) classification scale. A: Predicted vegetation patterns using the full dataset with the Random Forests Classifier; B: Predicted vegetation patterns using the FE dataset and the Support Vector Machine Classifier; C: Predicted vegetation patterns using the FS dataset with the Random Forests Classifier

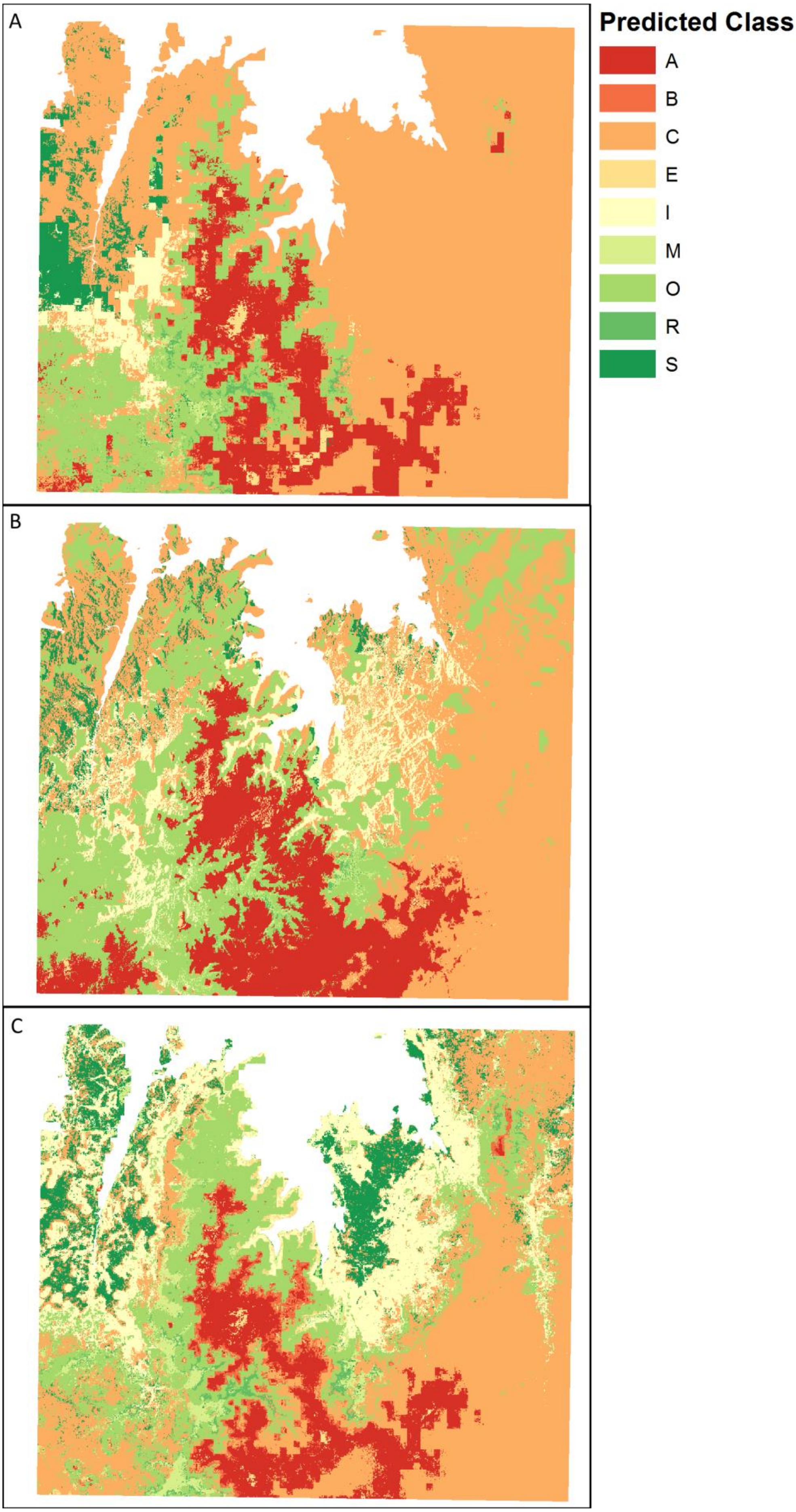
A
B
C
Predicted Class
A
B
C
E
I
M
O
R
S

Table S3. Summary table of the overall accuracy and kappa values of all model iterations

These represent the mean accuracy over all iterations per all combinations of factors. The numbers in brackets represent the average kappa value for that combination. The final columns and final rows represent the average accuracy and kappa for all runs.

	Full Dataset		FE Dataset		FS Dataset		Overall Mean (Classifiers)	
	kwongan	Woodland	kwongan	Woodland	kwongan	Woodland	kwongan	Woodland
Level A								
RF	41 (0.34)	50 (0.39)	32 (0.24)	45 (0.33)	42 (0.34)	52 (0.41)	38 (0.31)	49 (0.38)
SVM	35 (0.27)	20 (0.02)	35 (0.24)	48 (0.36)	35 (0.26)	50 (0.37)	34 (0.26)	39 (0.25)
CT	27 (0.20)	39 (0.27)	23 (0.15)	39 (0.27)	29 (0.21)	42 (0.30)	26 (0.19)	40 (0.28)
NN	22 (0.14)	13 (0.005)	24 (0.16)	39 (0.27)	25 (0.17)	49 (0.38)	24 (0.16)	33 (0.22)
Overall Mean (Level):	31 (0.24)	31 (0.17)	28 (0.20)	43 (0.31)	33 (0.25)	48 (0.37)	31 (0.23)	40 (0.28)
Level B								
RF	60 (0.47)	76 (0.50)	52 (0.38)	74 (0.48)	58 (0.45)	77 (0.53)	57 (0.43)	76 (0.51)
SVM	51 (0.36)	65 (0.03)	51 (0.36)	73 (0.46)	56 (0.43)	75 (0.50)	53 (0.38)	71 (0.33)
CT	46 (0.31)	58 (0.31)	42 (0.26)	69 (0.41)	50 (0.36)	69 (0.41)	46 (0.31)	65 (0.38)
NN	34 (0.16)	31 (0.03)	40 (0.24)	66 (0.38)	47 (0.31)	72 (0.48)	40 (0.24)	56 (0.29)
Overall Mean (Level):	48 (0.32)	57 (0.22)	47 (0.31)	71 (0.43)	53 (0.39)	73 (0.48)	49 (0.34)	67 (0.38)
Level C								
RF	85 (0.67)	85 (0.54)	80 (0.59)	83 (0.49)	85 (0.65)	84 (0.57)	83 (0.62)	84 (0.53)
SVM	82 (0.58)	78 (0.08)	82 (0.58)	84 (0.54)	84 (0.63)	87 (0.64)	83 (0.60)	83 (0.42)
CT	80 (0.52)	81 (0.46)	72 (0.40)	83 (0.49)	81 (0.58)	83 (0.52)	78 (0.50)	82 (0.49)
NN	65 (0.20)	30 (0.02)	74 (0.42)	82 (0.47)	80 (0.55)	82 (0.47)	73 (0.39)	65 (0.32)
Overall Mean (Level):	78 (0.49)	68 (0.28)	77 (0.49)	83 (0.50)	83 (0.60)	84 (0.55)	79 (0.53)	79 (0.44)
Overall Means:								
RF	62 (0.49)	70 (0.48)	55 (0.38)	67 (0.43)	62 (0.48)	71 (0.50)	60 (0.45)	69 (0.47)
SVM	56 (0.40)	54 (0.04)	55 (0.40)	68 (0.45)	58 (0.44)	71 (0.50)	58 (0.41)	64 (0.33)
CT	51 (0.35)	59 (0.35)	46 (0.27)	64 (0.39)	53 (0.38)	65 (0.41)	50 (0.32)	58 (0.35)
NN	40 (0.16)	24 (0.02)	46 (0.27)	62 (0.37)	51 (0.35)	68 (0.44)	46 (0.26)	51 (0.28)

Appendix S1. Matrices showing the results of all pairwise comparisons separated by Region and scale

All matrices use a consistent format representing the presence (and level) of significance for each comparison. For ease of interpretation, all matrices have been colour coded.

The significance levels are as follows:

*	Significant at <0.001
^	Significant at <0.01
#	Significant at <0.05
	Not significant

The matrices are labelled first by classifier then dataset as follows:

CT = Classification Tree

RF = Random Forests

SVM = Support Vector Machine

NN = Nearest Neighbour

DS1 = Predictions made using the Full dataset

DS2 = Predictions made using the FE dataset

DS3 = Predictions made using the FS dataset

Table 1:Pairwise matrix for the Kwongan vegetation at Level A

	CT_DS1	CT_DS2	CT_DS3	RF_DS1	RF_DS2	RF_DS3	SVM_DS1	SVM_DS2	SVM_DS3	NN_DS1	NN_DS2	NN_DS3
CT_DS1												
CT_DS2	^											
CT_DS3		*										
RF_DS1	*	*	*									
RF_DS2	*	*		*								
RF_DS3	*	*	*		*							
SVM_DS1	*	*	*	*		*						
SVM_DS2	*	*		*		*						
SVM_DS3	*	*	*	*		*						
NN_DS1	*		*	*	*	*	*	*	*			
NN_DS2			*	*	*	*	*	*	*			
NN_DS3			^	*	*	*	*	*	*	#		

Table 2:Pairwise matrix for the Kwongan vegetation at Level B

	CT_DS1	CT_DS2	CT_DS3	RF_DS1	RF_DS2	RF_DS3	SVM_DS1	SVM_DS2	SVM_DS3	NN_DS1	NN_DS2	NN_DS3
CT_DS1												
CT_DS2	#											
CT_DS3	#	*										
RF_DS1	*	*	*									
RF_DS2		*	*	*								
RF_DS3	*	*	*		*							
SVM_DS1	*	*		*		*						
SVM_DS2	*	*		*		*						
SVM_DS3	*	*	*				^	^				
NN_DS1	*	*	*	*	*	*	*	*	*			
NN_DS2	*		*	*	*	*	*	*	*	*		
NN_DS3		#	#	*	*	*	*	*	*	*	*	

Table 3: Pairwise matrix for the Kwongan vegetation at Level C

	CT_DS1	CT_DS2	CT_DS3	RF_DS1	RF_DS2	RF_DS3	SVM_DS1	SVM_DS2	SVM_DS3	NN_DS1	NN_DS2	NN_DS3
CT_DS1												
CT_DS2	*											
CT_DS3		*										
RF_DS1	*	*	*									
RF_DS2		*		*								
RF_DS3	*	*	*		*							
SVM_DS1		*		^		^						
SVM_DS2		*		#		#						
SVM_DS3	*	*	^		*							
NN_DS1	*	#	*	*	*	*	*	*	*			
NN_DS2	*		*	*	*	*	*	*	*	#		
NN_DS3		*		*		*	*		*	*	*	

Table 4: Pairwise matrix for the Woodland vegetation at Level A

	CT_DS1	CT_DS2	CT_DS3	RF_DS1	RF_DS2	RF_DS3	SVM_DS1	SVM_DS2	SVM_DS3	NN_DS1	NN_DS2	NN_DS3
CT_DS1												
CT_DS2												
CT_DS3												
RF_DS1	*	*	*									
RF_DS2	*	*		*								
RF_DS3	*	*	*		*							
SVM_DS1	*	*	*	*	*	*						
SVM_DS2	*	*	*		^	#	*					
SVM_DS3	*	*	*		*		*					
NN_DS1	*	*	*	*	*	*		*	*			
NN_DS2				*	*	*	*	*	*	*		
NN_DS3	*	*	*		*		*			*	*	

Table 5: Pairwise matrix for the Woodland vegetation at Level B

	CT_DS1	CT_DS2	CT_DS3	RF_DS1	RF_DS2	RF_DS3	SVM_DS1	SVM_DS2	SVM_DS3	NN_DS1	NN_DS2	NN_DS3
CT_DS1												
CT_DS2	*											
CT_DS3	*											
RF_DS1	*	*	*									
RF_DS2	*	*	*									
RF_DS3	*	*	*									
SVM_DS1	*	*	*	*	*	*						
SVM_DS2	*					^	*					
SVM_DS3	*	*	*				*					
NN_DS1	*	*	*	*	*	*	*	*	*			
NN_DS2				*	*	*		*	*	*		
NN_DS3	*					*	*			*	*	

Table 6: Pairwise matrix for the Woodland vegetation at Level C

	CT_DS1	CT_DS2	CT_DS3	RF_DS1	RF_DS2	RF_DS3	SVM_DS1	SVM_DS2	SVM_DS3	NN_DS1	NN_DS2	NN_DS3
CT_DS1												
CT_DS2												
CT_DS3												
RF_DS1												
RF_DS2												
RF_DS3												
SVM_DS1	#	*	*	*	*	*						
SVM_DS2							*					
SVM_DS3	*	#	^		#		*					
NN_DS1	*	*	*	*	*	*	*	*	*			
NN_DS2							^		*	*		
NN_DS3							^		*	*		
